# Characterization of Spontaneous Bone Marrow Recovery after Sublethal Total Body Irradiation: Importance of the Osteoblastic/Adipocytic Balance

**DOI:** 10.1371/journal.pone.0030818

**Published:** 2012-02-17

**Authors:** Géraldine Poncin, Aurore Beaulieu, Chantal Humblet, Albert Thiry, Kimimitsu Oda, Jacques Boniver, Marie-Paule Defresne

**Affiliations:** 1 Departments of Cytology & Histology and Pathological Anatomy (Giga-R), University of Liège, CHU-B23, Liège, Belgium; 2 Biochemistry, Niigata University, Graduate School of Medical and Dental Science, Niigata, Japan; National Cancer Institute, United States of America

## Abstract

Many studies have already examined the hematopoietic recovery after irradiation but paid with very little attention to the bone marrow microenvironment. Nonetheless previous studies in a murine model of reversible radio-induced bone marrow aplasia have shown a significant increase in alkaline phosphatase activity (ALP) prior to hematopoietic regeneration. This increase in ALP activity was not due to cell proliferation but could be attributed to modifications of the properties of mesenchymal stem cells (MSC). We thus undertook a study to assess the kinetics of the evolution of MSC correlated to their hematopoietic supportive capacities in mice treated with sub lethal total body irradiation. In our study, colony-forming units – fibroblasts (CFU-Fs) assay showed a significant MSC rate increase in irradiated bone marrows. CFU-Fs colonies still possessed differentiation capacities of MSC but colonies from mice sacrificed 3 days after irradiation displayed high rates of ALP activity and a transient increase in osteoblastic markers expression while pparγ and neuropilin-1 decreased. Hematopoietic supportive capacities of CFU-Fs were also modified: as compared to controls, irradiated CFU-Fs significantly increased the proliferation rate of hematopoietic precursors and accelerated the differentiation toward the granulocytic lineage. Our data provide the first evidence of the key role exerted by the balance between osteoblasts and adipocytes in spontaneous bone marrow regeneration. First, (pre)osteoblast differentiation from MSC stimulated hematopoietic precursor's proliferation and granulopoietic regeneration. Then, in a second time (pre)osteoblasts progressively disappeared in favour of adipocytic cells which down regulated the proliferation and granulocytic differentiation and then contributed to a return to pre-irradiation conditions.

## Introduction

Generally defined as a cellular microenvironment that controls the growth and differentiation of stem cells, hematopoietic stem cell (HSC) niches can be generated by several different cell types [1]. Among them, the osteopontin+/N-cadherin+/CD45- subpopulation of osteoblasts lining the bone surface has been shown to maintain a quiescent HSC pool [2], [3], [4], [5], [6], [7]. Endothelial cells form an alternative vascular niche where self-renewing HSC are maintained [8]. Interestingly, close to the osteoblastic and endothelial niches, sdf-1 abundant reticular (CAR) cells have been detected [9] and described as necessary for proliferation of HSC, lymphoid and erythroid progenitors as well as for maintenance of HSC in an undifferentiated state [10]. Adipocytes act as negative regulators of hematopoiesis; *in vitro*, they inhibit the differentiation of mature polynuclear neutrophils by blocking G-CSF production by macrophages via neuropilin-1 expression [11] and *in vivo*, they suppress hematopoietic progenitor proliferation [12]. In addition, they are able to enhance osteoblastic differentiation through leptin production [13], [14]. Finally, close to the osteoblastic niche, a population of fibroblast-like cells, called Western Bainton cells [15], characterized by membrane alkaline phosphatase (ALP) activity, form a network of cytoplasmic extensions around arteries and paratrabecular bone [16], [17]. Their topographic distribution is identical to granulocytic arrangement and it has been suggested that they influence granulopoiesis [18], [19].

Most of the cells of the bone marrow microenvironment may derive from a unique pluripotent stem cell, the mesenchymal stem cell (MSC), also called stromal stem cell [20], [21], [22]. MSC have the capacity both *in vivo* and *in vitro* to differentiate into osteoblasts, adipocytes, chondrocytes and muscle cells [23]. They are characterized by their capacity to form colonies *in vitro* (CFU-Fs) which reflects the mesenchymal progenitor proliferation capacities [24], [25].

Real time imaging studies of HSC in bone marrow *ex vivo* [26] and *in vivo* [27] suggest that the hematopoietic microenvironment dynamically changes its function under homeostasis and stressed conditions such as bone marrow radio-ablation for example. Many studies have focused on bone marrow hematopoietic regeneration after irradiation while paying with little attention to the recovery of cellular constituents of the microenvironment.

Several days after irradiation, the homeostasis of endothelial niche is altered [28] and the osteoblastic niche transiently expands [29]. After irradiation or chemotherapy or during the aging process, expansion of adipocytes correlates with myelosupression [30]. ALP+ activity is low after transplantation and associated with depressed hematopoiesis [31], whereas, in a reversible radio-induced aplasia model, an increase in the ALP network in the first 3 days after irradiation precedes hematopoietic regeneration which starts at day 5 [32]. This increase in ALP positive cells does not correlate with their proliferation and could be due to a shift in the MSC differentiation properties. We thus undertook a study to assess the kinetics of the evolution of MSC in mice treated with sub lethal total body irradiation in parallel with their hematopoietic supportive capacities.

## Results

### The proportion of mesenchymal stem cells increased in femoral bone marrows after irradiation: CFU-Fs assay

**Figure 1:**
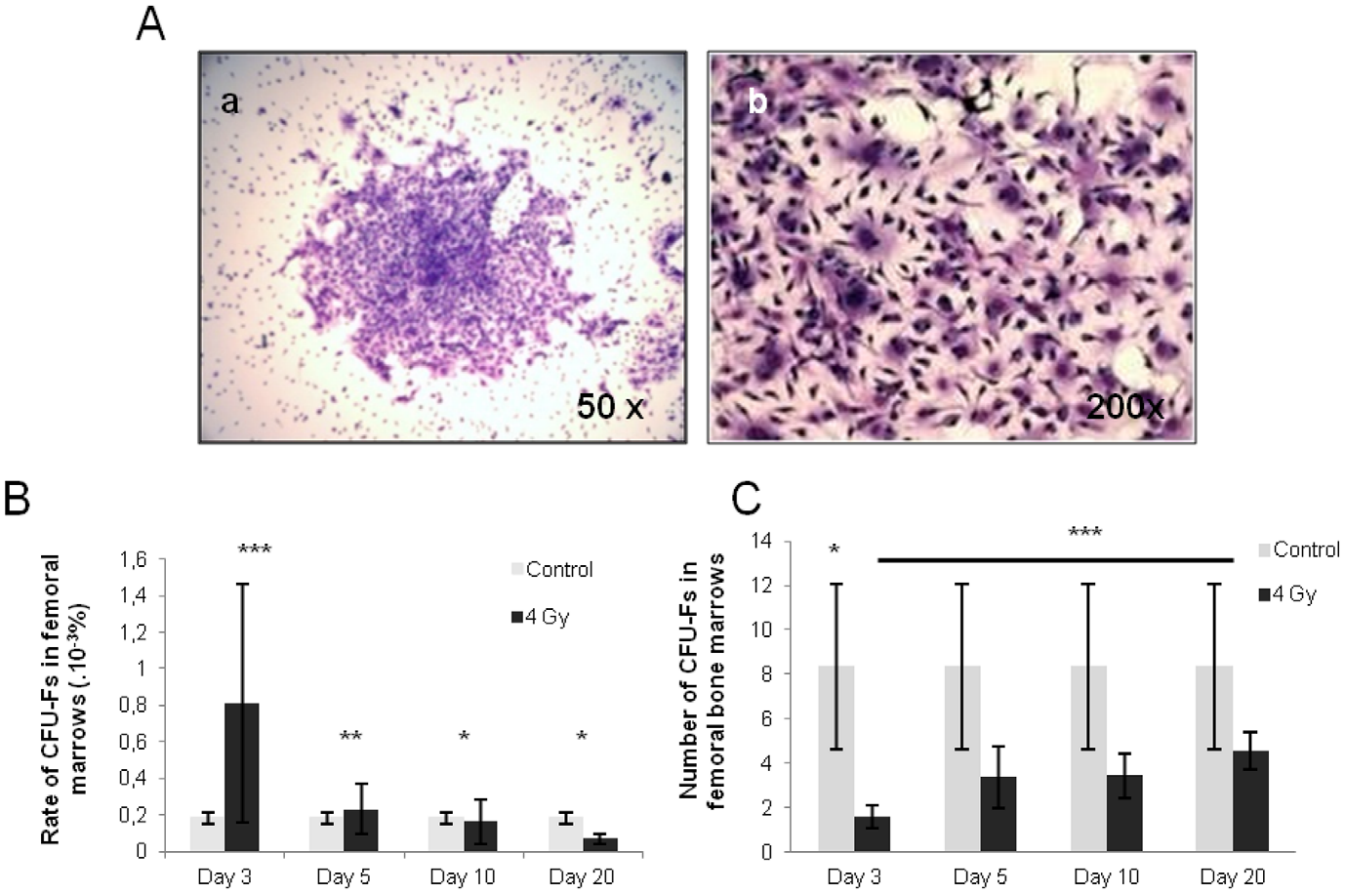
Percentages and absolute numbers of colony forming units-fibroblasts (CFU-Fs) in sublethally (4Gy) irradiated bone marrows. **A:** 10 days-cultured CFU-Fs colonies stained with May Grünmwald Giemsa (**a**: original magnification ×50 and **b**: original magnification ×200). **B:** Proportion of CFU-Fs in irradiated bone marrows *(black columns)* compared to controls *(grey columns)*.**C:** Absolute number of CFU-Fs in irradiated bone marrows *(black columns)* compared to controls (*grey columns*). Data are presented as mean values ± SEM of at least three independent experiments. ***, p<.001 ; **, p<.01 ; *, p<.05 as assessed by one way analysis of variance (ANOVA).

The presence of mesenchymal stem cells in irradiated bone marrows was evaluated using the CFU-Fs assay: 1.5 10^6^ total bone marrow cells were seeded as described in material and methods and were numbered after 10 days of culture (Fig. 1A).

The proportion of CFU-Fs in bone marrows was significantly increased when bone marrow cells were harvested at day 3 after irradiation (0.81 10^−3^±0.66 10^−3^%) (Fig. 1B) in comparison to controls (0.18 10^−3^±0.03 10^−3^%) (p<.001) while their absolute number decreased significantly (from 8.37±3.72 cells/femur in controls to 1.59±0.49 cells/femur at day 3) (p<.05) (Fig. 1C). Thereafter, at days 5, 10 and 20, the percentage of CFU-Fs returned to values similar to that observed in control bone marrows (0.23 10^−3^±0.14 10^−3^% (p<.01); 0.16 10^−3^±0.12 10^−3^% (p<.05) and 0.07±0.03 10^−3^% respectively) (p<.05) (Fig. 1B) as their absolute number progressively increased (4.58±0.82 at day 20, p<.001) (Fig. 1C).

Although their relative radiosensitivity, the proportion of mesenchymal stem cells in femoral bone marrow increases transiently 3 days after a 4 Gy total body irradiation.

### CFU-Fs exhibited phenotypic and differentiation capacities similar to MSCs

We sought to identify the nature of CFU-Fs cells. CFU-Fs were first analyzed by immunohistochemistry for a panel of antigens. Like mesenchymal stem cells, most of the cells in these colonies expressed CD106 and Sca-1. They were negative for CD45 and CD11b (results not shown). But, contrary to MSC, they were no longer able to form secondary colonies.

Osteoblastic differentiation of CFU-Fs from control mice and from irradiated mice sacrificed at day 3 after irradiation into osteoblasts was induced *in vitro* by treating cells with low concentrations of ascorbic acid, dexamethasone and B-glycerophosphate. CFU-Fs cultured with this osteoinductive medium showed a progressive change in morphology from spindle-shaped to cuboidal accompanied by an hydroxyapatite deposition revealed by alizarin red staining at day 21 suggesting that CFU-Fs are able to differentiate into mature osteoblasts *in vitro*.

To induce adipocytic differentiation, we selected a medium containing rabbit serum, dexamethasone and insulin to obtain intracellular lipid droplets stained by oil red O. These vacuoles appeared at day 8 and reached a maximum at day 14. These results suggest that most CFU-Fs have the potential to differentiate into adipocytes. (results not shown).

When cultured in appropriate mediums, adipocytic and osteogenic differentiation capacities of CFU-Fs colonies from normal and irradiated mice are equivalent.

### Alkaline phosphatase expression, a common osteoblastic marker, increased in CFU-Fs from irradiated mice

**Figure 2:**
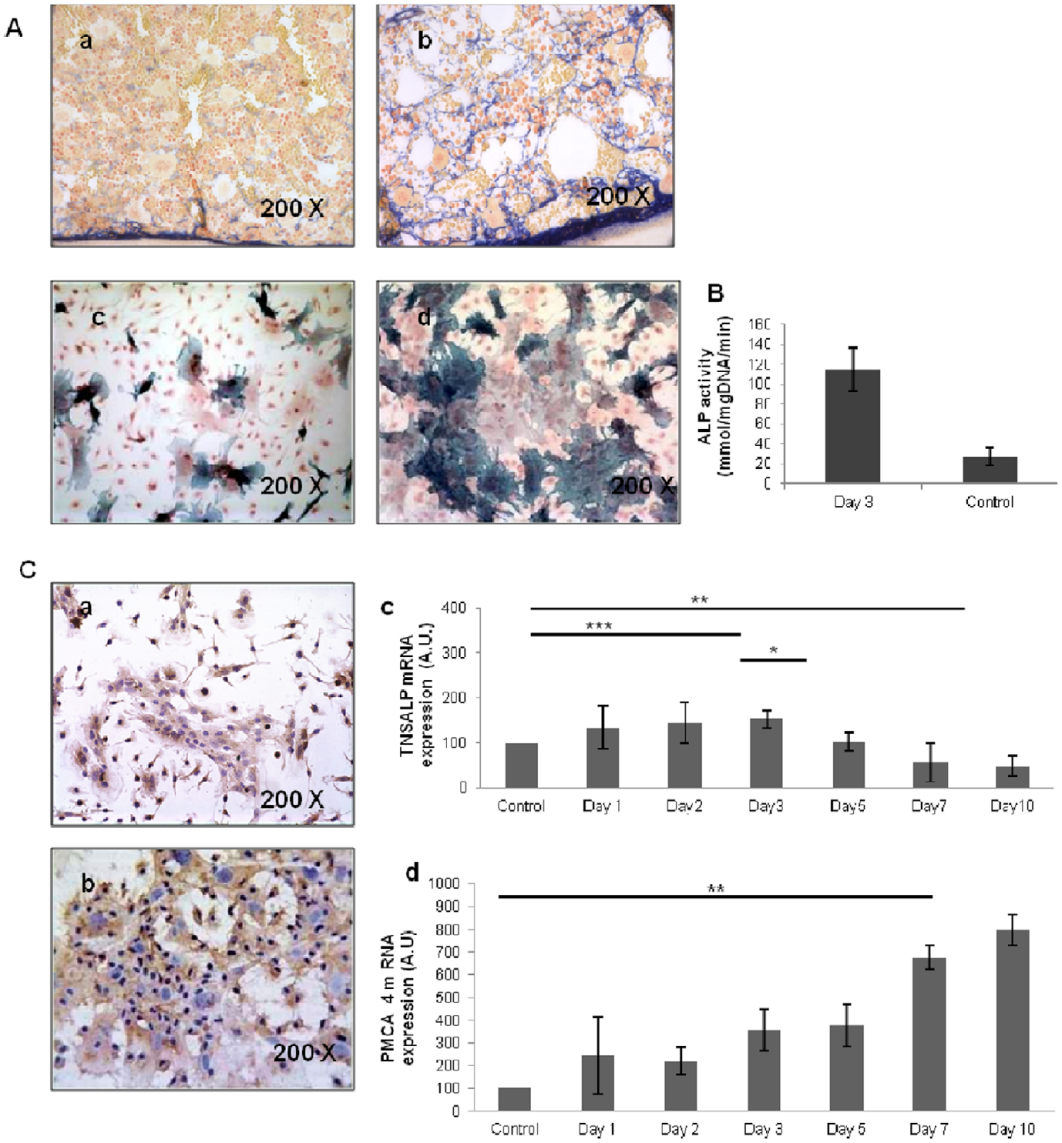
Alkaline phosphatases (ALPs) in CFU-Fs. **A:** Morphologic observation of ALP activity *(in blue)* both in *in situ* bone marrows from controls (**Aa**) or irradiated (**Ab**) mice (day 3 after irradiation) and in 10 days-cultured CFU-Fs from controls (**Ac**) or irradiated (**Ad**) mice (original magnification ×200). **B:** Comparison of the ALP activity quantified by colorimetric reaction in 10 days-cultured CFU-Fs from controls and day-3 irradiated mice. **C**: Expression of Tissue non specific alkaline phosphatase (TNSALP) and Plasma membrane calcium ATPase (PMCA) in 10 days-cultured CFU-Fs : CFU-Fs are positive for TNSALP (**Ca**) and PMCA (**Cb**) isoforms as tested by immunohistochemistry (original magnification: ×200). mRNAs for the two isoforms are increased soon after irradiation (**Cc** : TNSALP mRNA and **Cd** : PMCA mRNA). Data are presented as mean values *±* SEM of at least three independent experiments. ***, p<.001; **, p<.01 ; *, p<.05 as assessed by one way analysis of variance (ANOVA).

*In situ* observations showed changes in the distribution of ALP positive cells in the bone marrow; mainly located near the endosteal surface in basal conditions (Fig. 2Aa), they extended cytoplasmic processes throughout the entire bone marrow 3 days after irradiation (Fig. 2Ab).

Cells of CFU-Fs, from irradiated or non-irradiated mice, also presented this ALP activity. However, ALP activity was detected in 14% of the cells forming CFU-Fs colonies in control conditions (Fig. 2Ac) , whereas this rate reached 46% in colonies obtained from mice sacrificed at day 3 after irradiation (Fig. 2Ad). Thereafter this percentage decreased progressively to reach 27.6% at “day 5”, 22.7% at “day 10” and 7.7% at “day 20” as hematopoietic regeneration took place. This evolution was similar to that observed *in situ*. ALP activity quantification with p-nitrophenylphosphate confirmed a significantly more important activity in CFU-Fs from irradiated mice (11.14 10^4^+2.16 10^4^ nmol/(mg DNA)/min)) compared to controls (2.65 10^4^+0.86 10^4^ nmol/(mg DNA)/min) (p<.001) (Fig. 2 B). No ALP activity was detected in cell culture supernatants.

Immunohistochemistry revealed that the ALP activity expressed *in situ* in irradiated bone marrows and in CFU-Fs was due to the presence of both Tissue Non Specific Alkaline Phosphatase (TNSALP) and Plasma Membrane Calcium ATPase (PMCA) isoforms as described by Nakao [33] (Fig. 2Ca, Cb). RT PCR experiments on *in vitro* irradiated CFU-Fs revealed an increase in TNSALP mRNA expression since the first day after irradiation that reached 153.16+19.04% of the control values at day 3 (p<.001). This rate progressively decreased since day 5 to reach 48.76+21.45% at day 10 (p<.01) (Fig. 2Cc) while a progressive increase in PMCA RNA rate was observed (Fig. 2Cd).

### Irradiation of CFU-Fs induced a transient increase in osteogenic markers and a decrease in adipocytic ones

**Figure 3:**
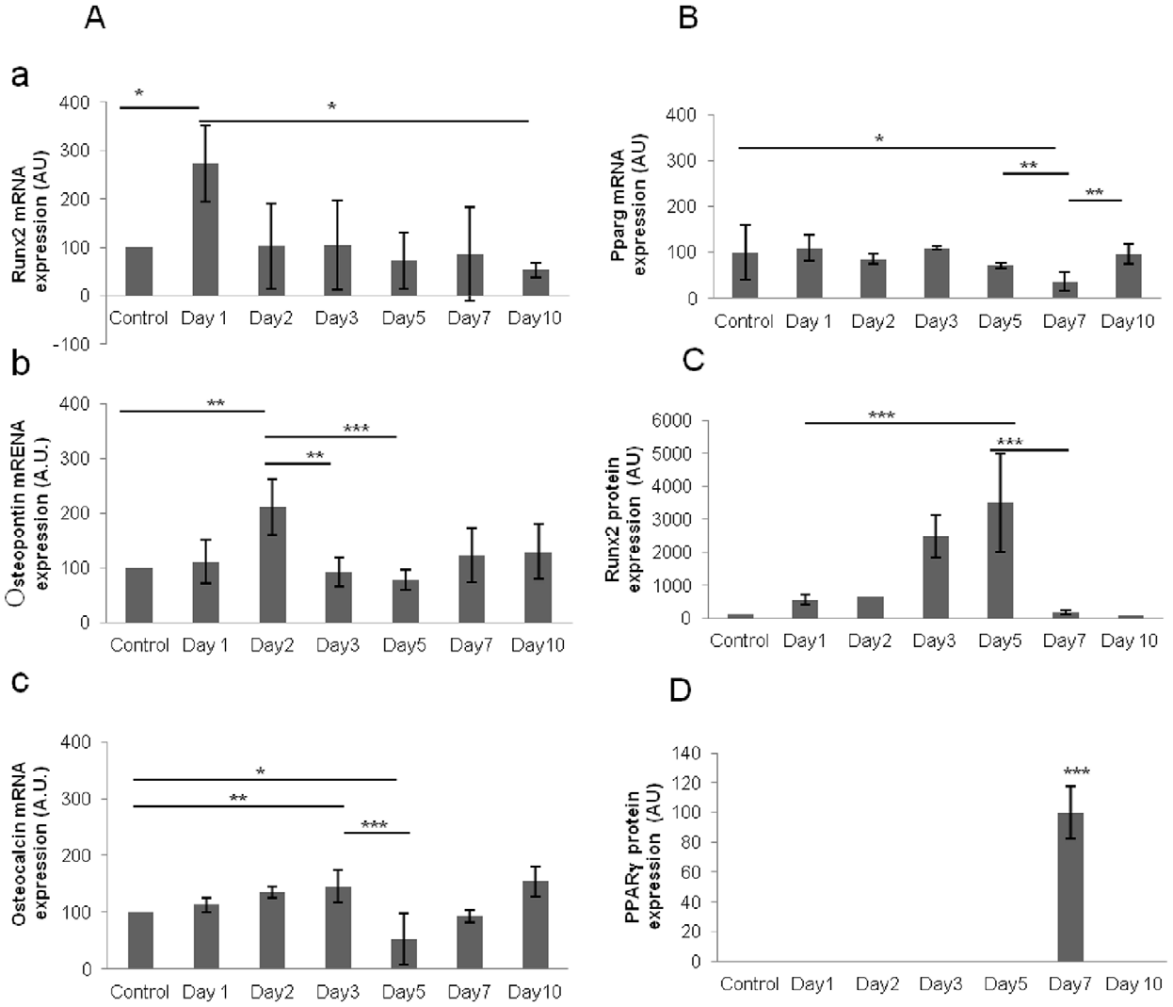
Expression of osteogenic and adipocytic markers in CFU-Fs after irradiation. **A**: Kinetics of expression of osteoblastic markers mRNAs (**Aa:** Runx2, **Ab:** Osteopontin and **Ac:** Osteocalcin) after irradiation. **B:** Kinetics of expression of the adipocytic marker PPARγ mRNA after irradiation. **C:** Kinetics of expression of the adipocytic protein PPARγafter irradiation. **D:** Kinetics of expression of the osteoblastic protein Runx2after irradiation. Data are presented as mean values ± SEM of at least three independent experiments. ***, p<.001; **, p<.01; *, p<.05 as assessed by one way analysis of variance (ANOVA).

RT PCR experiments on *in situ* irradiated CFU-Fs revealed expression of osteogenic and adipocytic markers (Fig. 3).

As compared to control values, we observed a significant increase in runx2 mRNA expression since the first day after irradiation (272.92±77.66%) (p<.05) (Fig. 3Aa) followed, (at day 2) by an increase in osteopontin mRNAs (210.38±50.26%) (p<.01) (Fig. 3Ab) and, at day 3, in osteocalcin mRNAs (145.45±27.98%) (p<.01) (Fig. 3Ac) suggesting a transition into the osteoblastic lineage. Since the second day after irradiation, runx2 mRNA expression started to decrease and reached 52.99±14.08% at day 10 (p<.05). A similar but later decrease has also been observed in osteocalcin (52±45.2% at day 5) (p<.05) and osteopontin (77.72±18.27% at day 5) (p<.001) mRNAs expression.

Pparγ mRNA, a maker of adipocytic differentiation, decreased after irradiation to 36.74±22% of the control level at day 7 (p<.05) suggesting a reduction of adipocytic differentiation.

These results were confirmed by western immunoblotting experiments a progressive increase of Runx2 protein was observed until day 5 (3498.67+1484.25%) (Fig. 3C) and PPARγ suddenly appeared at day 7 after irradiation (100±17.73%) (Fig. 3D).

### Evolution of the peripheral blood and bone marrow hematopoietic cells after irradiation

**Figure 4:**
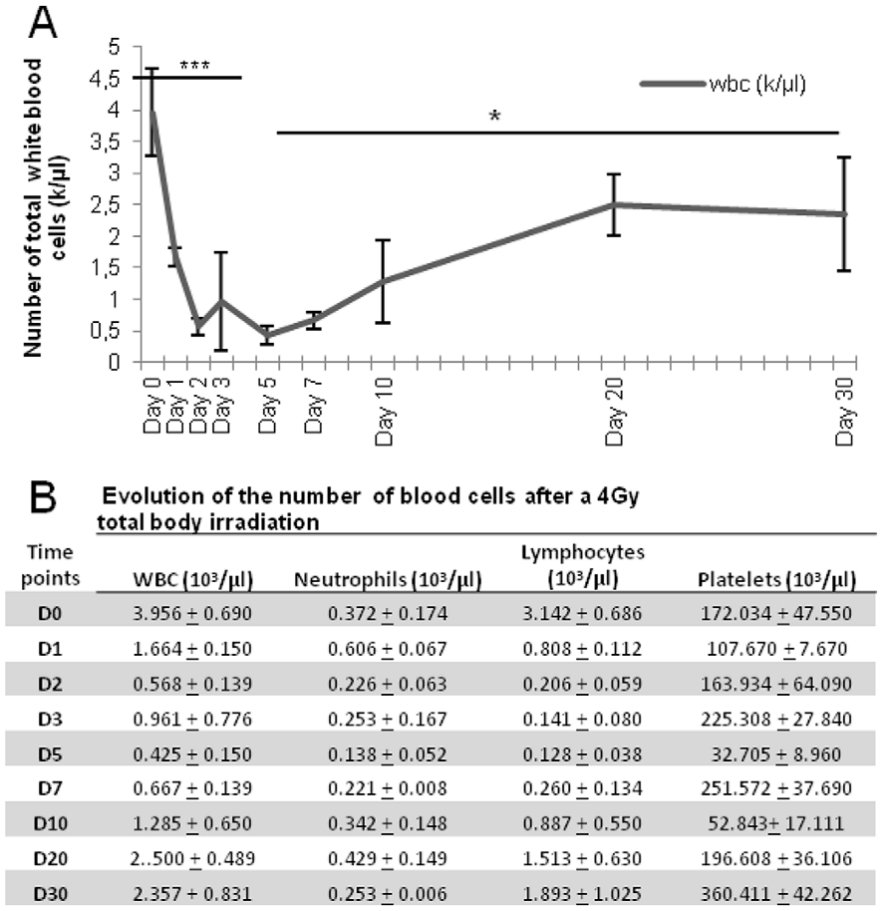
Evolution of white blood cells (WBCs) numbers, of neutrophils, lymphocytes and platelets in peripheral blood after a 4Gy total body irradiation. **A:** Evolution of WBCs in peripheral blood after irradiation. **B**: Number of circulating cells (WBC, neutrophils, lymphocytes and platelets) at different time points after irradiation. Data displayed as mean values ± SEM of at least three independent determinations. ***, p<.001; **, p<.01; *, p<.05 as assessed by one way analysis of variance (ANOVA).

A 4 Gy irradiation induced a significant decrease in the number of total white blood cells (WBC) (from 3.95±0.69 10^3^ cells/µl in controls to 0.425±0.15 10^3^ cells/µl at day 5, p<.001) (Fig. 4A). Since day 7, a spontaneous and progressive restoration was observed and the number of total WBC reached 2.36±0.83 10^3^ cells/µl at day 30. The levels of neutrophils, lymphocytes and platelets followed the same kinetics (Fig. 4B).

**Figure 5:**
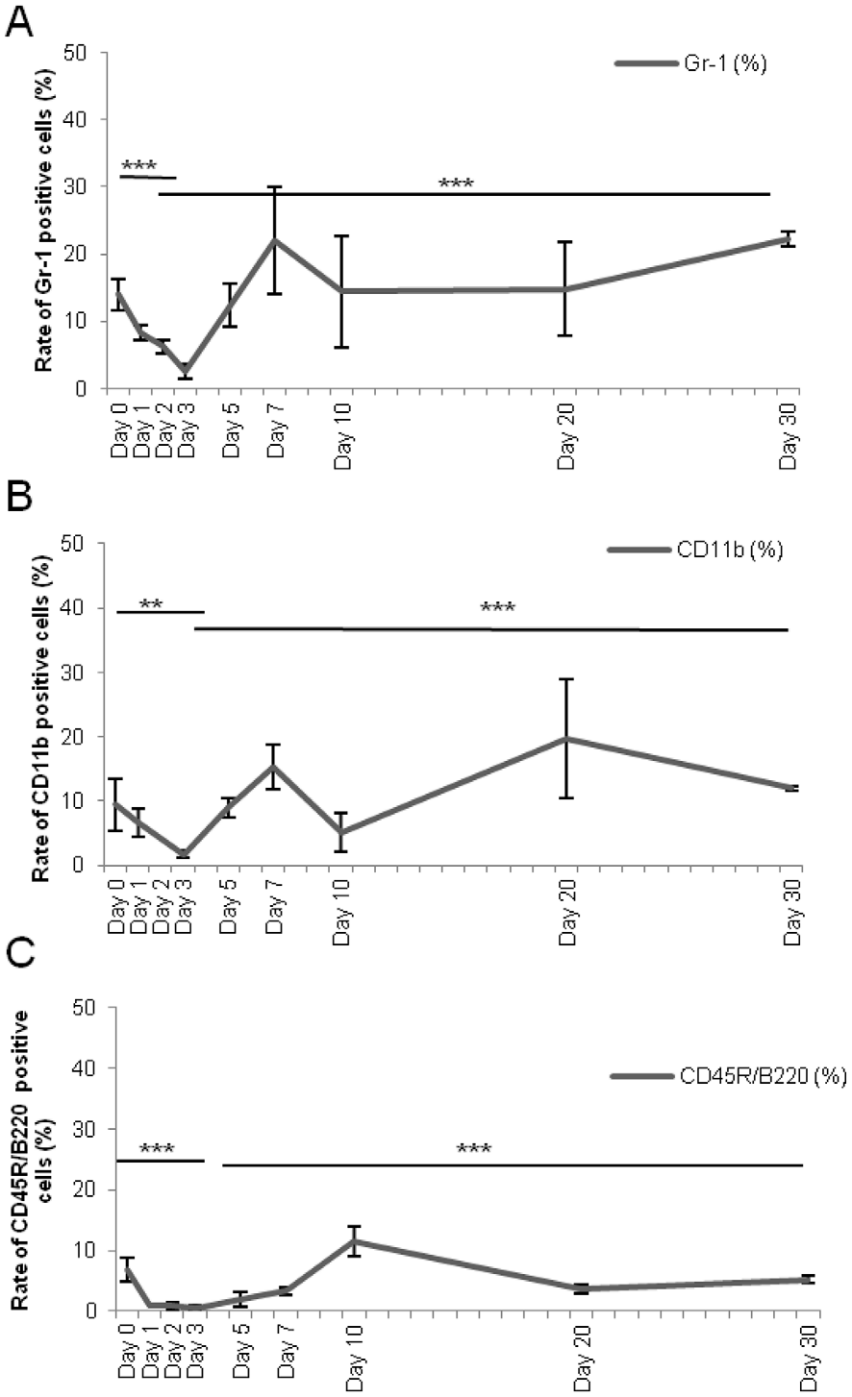
Evolution of the percentage of hematopoietic precursors in sublethally (4Gy) irradiated bone marrows. **A:** Granulocytes (Gr-1+), **B:** Monocytes (CD11b+), **C:** Lymphocytes (CD45R/B220+). Data are presented as mean values ± SEM of at least three independent experiments. ***, p<.001 ; **, p<.01 ; *, p<.05 as assessed by one way analysis of variance (ANOVA).

The same evolution was observed in bone marrow: irradiation, induced a significant decrease in the rate of Gr-1+ cells (Fig. 5A) (from 14±2.35% to 2.5±1.05% at day 3, p<.001) of CD11b (Fig. 5B) cells (from 9.38±3.94% to 1.70±0.56% at day 3, p<.001) and of CD45R/B220 cells (Fig. 5C) (from 6.92±1.95% to 0.43±0.40% at day 3, p<.001). Thereafter a spontaneous and progressive regeneration, for each lineage was observed from day 5 to day 30.

4Gy irradiation induced depletion in circulating total white blood cells (WBC) associated to a bone marrow monocytic (CD11b), lymphocytic (CD45R/B220) and granulocytic (Gr-1) reduction. Spontaneous regeneration started at day 5.

### CFU-Fs from control and irradiated mice sustained *in vitro* hematopoietic differentiation, proliferation and survival

To investigate the possible role of CFU-Fs from irradiated mice in hematopoietic regeneration, we tested the proliferation, the capacity to form colonies and the phenotype of hematopoietic precursors co-cultured with CFU-Fs from day 3 irradiated or control mice. In all experiments, 0.2 10^6^ hematopoietic progenitors from control mice were incubated in direct contact or in transwell conditions with CFU-Fs obtained from control or day 3-irradiated mice.

After the first day of co-culture, close contacts were observed between haematopoietic precursors and CFU-Fs. Hematopoietic cells cannot be harvested by a simple wash, so they must be treated by PBS-EDTA at 37°C for 10 min. After tryptan blue exclusion, living haematopoietic cells were counted after 7 days of co-culture. The mean number of harvested hematopoietic cells was significantly higher after co-culture with CFU-Fs from mice killed 3 days after irradiation (0.36 10^6^+0.07 10^6^) than with CFU-Fs from control animals (0.15 10^6^+0.01 10^6^) (p<.001).

**Figure 6:**
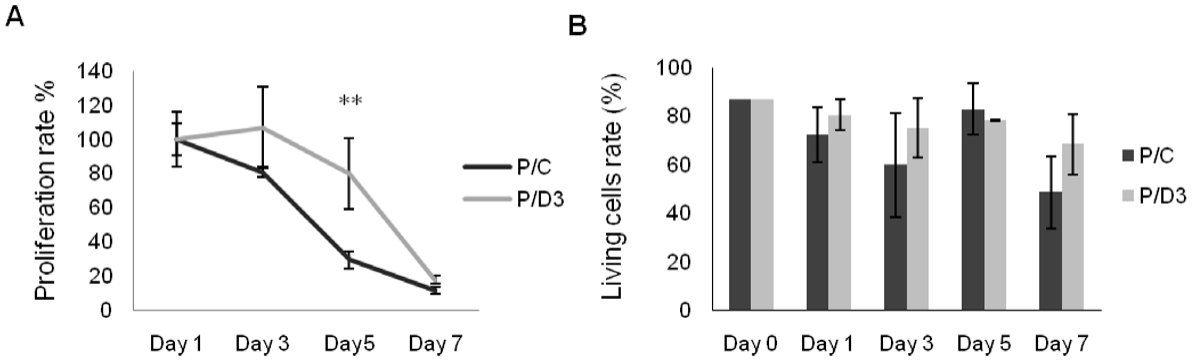
Proliferation and survival of hematopoietic precursors in co-culture with CFU-Fs from irradiated (*grey*) and non-irradiated (*black*) bone marrows. **A:** Proliferation of hematopoietic precursors tested by ^3^H incorporation. **B:** Survival of hematopoietic precursors tested by Annexin-PI labeling. Data are presented as mean values ± SEM of at least three independent experiments. **, p<.01 as assessed by one way analysis of variance (ANOVA).

^3^H incorporation tests demonstrated that this difference was due to an increase in proliferation: the values obtained in co-culture with CFU-Fs from mice sacrificed 3 days after irradiation were significantly different from those obtained in co-culture with control CFU-Fs (11951±3073.8 cpm and 4394±785.6 cpm respectively) (p<.01) (Fig. 6A).

No significant differences in cell survival, as tested by annexin-PI were observed when precursors are co-cultured on CFU-Fs from control or irradiated mice (Fig. 6B).

**Figure 7:**
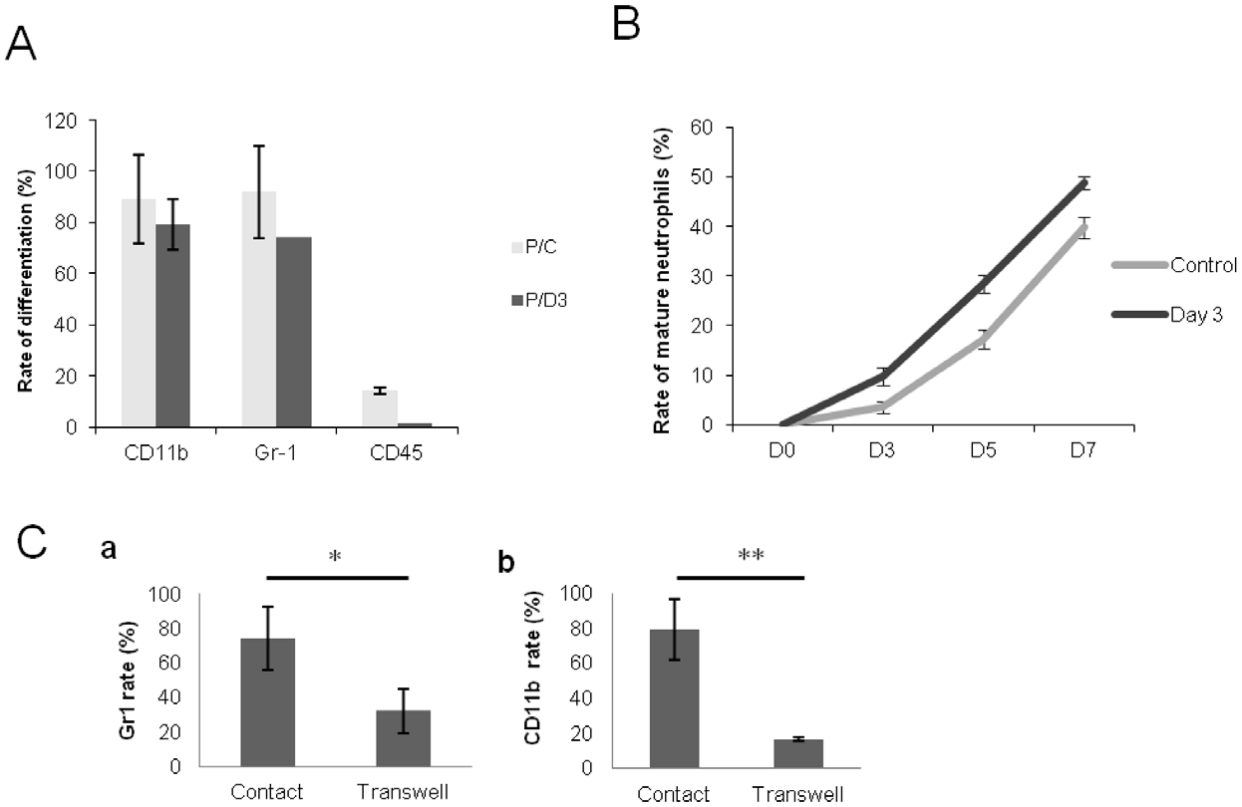
Differentiation of hematopoietic precursors in direct contact with CFU-Fs from irradiated (*black*) and non-irradiated (*grey*) bone marrows. **A:** Immunophenotyping shows a myelo-monocytic (CD11b), a granulocytic (Ly-6G) and a B-lymphoid (CD45R/B220) engagement of hematopoietic precursors in lineages. **B:** The differentiation of mature neutrophils during the co-culture assay occurs earlier when precursors are co-cultured on “day 3” CFU-Fs. **C:** Inhibition of the contact in transwell conditions induces a significant reduction in the differentiation of the Gr1+ (**Ca**) and CD11b+ (**Cb**) lineages. Data are presented as mean values ± SEM of at least three independent experiments. **, p<.01; *, p<.05 as assessed by one way analysis of variance (ANOVA).

After 7 days of co-culture, hematopoietic harvested cells were not yet able to form CFU colonies in methylcellulose. Immunophenotyping indicated a myelo-monocytic (CD11b) (89.5±17.34% in controls, 79.19±9.83% in Day 3), a granulocytic (Ly-6G) (91.9±18.24% in controls, 74.16% in day 3) and a B-lymphoid (CD45R/B220) (14.3±1.28% in controls, 1.41±0.27% in day 3) engagement in lineages (Fig. 7A). Although the proportion of cells engaged in each lineage was not significantly different, the maturation started at different time points according the conditions of co-culture; the appearance of mature neutrophils during the co-culture assay occurred earlier when precursors are co-cultured on “day 3” CFU-Fs (Fig. 7B).

### Contact between CFU-Fs and hematopoietic precursors is required for hematopoietic differentiation

To understand the nature of the relation between CFU-Fs and hematopoietic progenitors and the importance of the contact in hematopoiesis, we inhibited the contact between these two populations in co-culture: they were separated by a 4 µm semi-permeable membrane, only soluble factors and cytokines were thus able to travel between the two compartments.

We did not observe any significant difference in the survival and the proliferation rate between control and transwell conditions. However, inhibition of the contact induced a significant reduction of the differentiation rate both for Gr-1 (from 74.15±18.23% in controls to 32.13±12.91% in transwell conditions, p<.05) (Fig. 5Ca) and for CD11b lineages (from 75.19±17.34% in controls to 16.63±9.87% in transwell conditions, p<.01) (Fig. 7Cb).

### Neuropilin 1 (NP1), a molecule implicated in the inhibition of neutrophils maturation is down regulated in irradiated CFU-Fs

**Figure 8:**
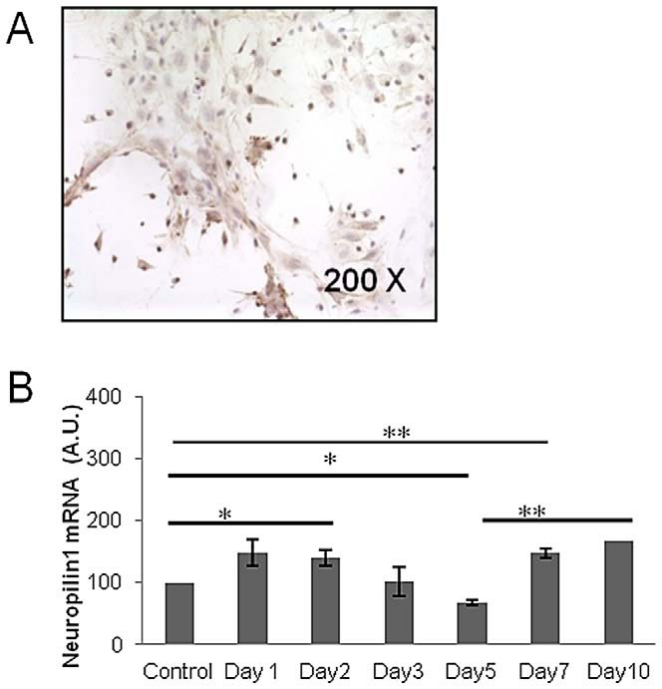
Expression of neuropilin-1 *in* CFU-Fs. **A:** Immunohistochemistry for Neuropilin-1 expression in irradiated mice CFU-Fs (magnification ×200). **B:** Evolution of neuropilin-1 mRNAs in *in vitro*-irradiated CFU-Fs. Data are presented as mean values ± SEM of at least there independent experiments. **, p<.01; *, p<.05 as assessed by one way analysis of variance (ANOVA).

NP1 was shown to be implicated in the inhibition of neutrophil maturation *in vitro*. 48 h after irradiation, its expression transiently increased (140.27±12.79, p<.01) (Fig. 6A) Thereafter and until day 5 after irradiation, the mRNAs levels were significantly lower than those measured in control conditions (67.16±5.01%, p<.05) (Fig. 8B).

### The stromal cell-derived factor 1 (sdf-1), a regulator of hematopoiesis and stem cell homing, is increased after irradiation

**Figure 9:**
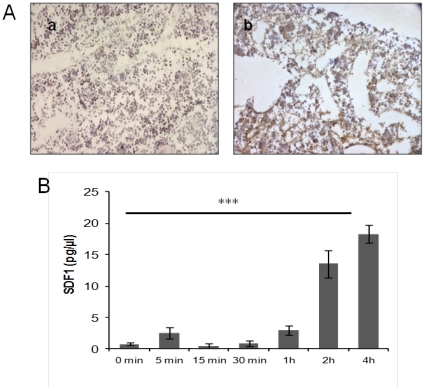
SDF-1 expression in irradiated bone marrows and CFU-Fs. **A:** Labelling with anti-sdf1 antibodies is more intense in bone marrows from day-1 irradiated mice (**Ab**) compared to control ones (**Aa**) (original magnification ×200). **B:** Elisa quantification of sdf-1 in the supernatant of irradiated CFU-Fs showed an increase in sdf-1 until 4 hours after irradiation. **C:** The level of mRNA expression is not significantly different in control and irradiated CFU-Fs. Data are presented as mean values ± SEM of at least three independent experiments. ***, p<.001 as assessed by one way analysis of variance (ANOVA).

Elisa quantifications on the supernatant of irradiated CFU-Fs showed an sizeable increase in sdf-1 until 4 hours after irradiation in comparison to basal conditions (respectively 18.31±1.43 pg/µl and 0.81±0.25 pg/µl , p<.001) (Fig. 9B) which corroborates *in situ* observations (Fig. 7 A). This increase was not correlated with an increase in sdf-1 mRNA (Fig. 9C) which suggests release of endogenous sdf-1 stocks.

## Discussion

Many studies have described the bone marrow haematopoietic regeneration post chemotherapy and bone marrow transplantation [34]. Stromal regeneration and particularly the evolution of hematopoietic niches haven't yet been extensively described. Such an analysis could be helpful to understand the function of these cells in the support of haematopoiesis and their role in pathological conditions.

This work was designed to analyze the recovery of the bone marrow stromal niches, within a model of radio-induced medullary aplasia. This could help to better understand the influence and the properties of the hematopoietic niches components and particularly (pre)osteoblasts and (pre)adipocytes in physiological and pathological conditions.

Previous experiments have shown an increase in the bone marrow ALP network, 3 days after sublethal irradiation, preceding spontaneous hematopoietic regeneration [32]. This work reported an increase in the number of ALP positive cells in adult mice bone marrow cavity although BrdU incorporation tests *in vivo* demonstrated that this increase wasn't due to the ALP positive cells proliferation. Our immunohistochemical analysis revealed a specific pattern of ALP positive cells repositioning after irradiation. In normal conditions, the majority of ALP positive cells reside in close proximity to bone and to sinusoidal vessels. As we report here, irradiation induced a shift in the location of ALP positive cells to the whole bone marrow cavity, where they maintained close contacts with hematopoietic cells suggesting interactions between these 2 cells types. These observations raised some questions: Do these cells come from outside the bone marrow? Are these cells recruited among other bone marrow stromal cells? One hypothesis is that they differentiate from mesenchymal stem cells (MSC).

To address these questions, we analysed the evolution of CFU-Fs which reflect the presence of mesenchymal stem cells in the bone marrow [24], [35] and observed, in irradiated bone marrows, that their absolute number decreased but that their proportion increased in parallel to the number of ALP positive cells *in situ*. Although they proliferate after bone marrow disturbances *in vitro* [36], in physiological conditions mesenchymal stem cells are mostly quiescent. These results indicate that they are relatively radioresistants in the case of a 4 Gy irradiation and suggest that they proliferate during bone marrow recovery.

CFU-Fs obtained from irradiated bone marrows lacked CD11b and CD45 expression and expressed CD106 and Sca-1. They were able to differentiate into adipocytes and osteoblasts under appropriate stimulating conditions. These properties are similar to those of MSC [37], [38].

However, as compared to MSC or to CFU-Fs isolated from normal bone marrow, CFU-Fs isolated 3 days after irradiation contained a higher proportion of cells expressing alkaline phosphatase, a common osteoblastic differentiation marker [39]. This was confirmed by an increase of ALP activity measured by colorimetry. Further characterization showed a spontaneous increase in the expression of osteoblastic differentiation markers like runx2, osteocalcin, osteopontin, TNSALP and PMCA in the first 3 days following irradiation and a decrease in adipocytic markers expression (pparγ). These modifications were reversible: RunX2 started to decrease after 2 days, whereas osteocalcin, osteopontin and TNSALP returned to control values respectively at day 5, day 3 and day 5 after irradiation. These observations suggest a disturbance in the osteoblast/adipocyte balance: until day 3 after irradiation bone marrow stromal cells seemed to engage within the osteoblastic lineage in a reversible way and to return to a less differentiated stage thereafter.

Some cases of such imbalances have already been reported in stress conditions as well as in pathological conditions : hypoxia has been described to induce proliferation of mesenchymal stem cells and an increase in the alkaline phosphatase positive flattened cell population [40]; the exposition to electromagnetic fields promotes osteoblastic differentiation and inhibit adipocyte formation [41] ; the alkaline phosphatase activity is increased in spleen and lymph nodes from leukemic mice [42] and increased marrow adiposity is linked with aging, bone loss and osteoporosis [43].

(Pre)osteoblasts were previously identified as crucial components of the hematopoietic supportive stroma [3], [5], [44], [45], [46]. To investigate the influence of (pre)osteoblastic cells on hematopoiesis, we co-cultured CFU-Fs from day 3 irradiated or non-irradiated mice with hematopoietic precursors (ckit+Lin-) and compared the results obtained *in vitro* with hematopoietic regeneration *in vivo*.

Proliferation of normal haematopoietic precursors was greatly enhanced in co-cultures with CFU-Fs obtained from mice sacrificed 3 days after irradiation as compared to cultures with CFU-Fs from normal bone marrow. Previous observations have also underlined the importance of (pre)osteoblastic cells in hematopoietic stem cell proliferation and mobilization [44]. CFU-Fs from normal and irradiated mice were unable to maintain immature haematopoietic cells (ckit+lin-) in their precursor stage of differentiation as tested by their capacities to form hematopoietic colonies *in vitro*. Both of them induced their differentiation into granulocytes (Gr-1), B-cells, macrophages and platelets. These observations correlate with the hematopoietic recovery observed in vivo both in bone marrow and in the peripheral blood. The recovery of granulocytes is in agreement with morphological observations suggesting a role of alkaline phosphatase positive cells in granulopoiesis [16], [18]. However it cannot be excluded that macrophages, initially present in the CFU-Fs cultures contribute to this effect: indeed they are considered as an essential component of the hematopoietic microenvironment [47].

Although the induction of differentiation of hematopoietic precursors into granulocytes was similar whatever the condition, mature polynuclears neutrophils appeared earlier when hematopoietic precursors were cultured with CFU-Fs derived from irradiated mice. This may be partly explained by a rapid decrease in neuropilin-1 after irradiation, a molecule well known to inhibit the maturation of polynuclear neutrophils by the inhibition of G-CSF production [11].

Among molecules involved in stem cell recruitment, CXCL12 (sdf-1)-CXCR4 axis has been described to take an important part in hematopoietic regeneration after irradiation or chemotherapy [48], [49]. Principally produced by (pre)osteoblasts [50], [51] and endothelial cells [52] sdf-1 is considered as an important regulator of hematopoietic proliferation and myelopoiesis [53], [29]. The rapid increase of sdf-1 after irradiation both *in vitro* and *in vivo* can be correlated with its role in the osteoblastic niche: first, by promoting osteoblastic differentiation [54] and secondly by recruiting hematopoietic precursors into the niche. These interactions are reinforced by the increase in ostepontin expression [55]. Recently CXCL12 abondant reticular cells (CAR) have been decribed as cells producing high amounts of sdf-1 and contributing to the regulation of hematopoiesis [56]. ALP positive (pre)osteoblasts and CAR cells present structural similarities, thus we cannot exclude that they belong to the same cell type.

Following all these observations, we hypothesize that the primary effect of a 4Gy rate of total body irradiation is to enhance (pre)osteoblast differentiation from MSC to favor, in a first time, hematopoietic precursors proliferation, granulopoietic and lymphoid regeneration and to accelerate mature polynuclear neutrophil differentiation. In a second time (pre)osteoblasts progressively disappeared in favor of adipocytic cells which down regulate the proliferation and differentiation rate and contribute to a return to pre-irradiation condition.

## Materials and Methods

### Animals

6 week-old C57BL/6 mice were used. Initially obtained from M Lieberman (Stanford University, Stanford, CA, USA), this strain was then raised in the Animal House of the University of Liège. All of our mice are housed in SPF zone in individual ventilated cages and are changed in a hood using the 2-forcep technique and Virkon ®. Their bedding is autoclaved, food is irradiated, and water is acidified. The experimental work was conducted according to the procedures outlined in the Law for Care and Use of Laboratory Animals (Arrêté Royal, 14th November 1993, Belgium). All this experiences were conducted with the approbation of the board of animal ethics from the University of Liège (folder 1088).

### Irradiation procedures

A total of 4 Gy acute whole body irradiations (1.15 Gy/min) was administered to mice with a gamma-ray machine (Gammacell 40 exactor, 137Cs, 662 KeV, Nordion, Ottawa, Canada, http://www.mds.nordion.com). Mouse whole body irradiation was carried out in a special irradiation box, which can hold 10 mice together.

### Blood and bone marrow cells

Mice were sacrificed under anesthesia (Isoflurane, Forene©, Abbott, Wavre, Belgium, http://www.abbott.be) by cervical dislocation at different time points after irradiation (1, 3, 5, 7, 10 and 20 days). The total peripheric blood was harvested and their bone marrow cells were flushed from the femurs into phosphate buffer saline (PBS) using a syringe and a 21-Gauge needle. Residual bone marrow red blood cells were lysed by incubation in a hypotonic solution of ammonium chloride. Cells counts were performed and viability was determined by tryptan blue (Invitrogen, Liege, Belgium, http://www.invitrogen.com) exclusion.

Total blood counts were performed on an hemocytometer (Cell Dyn 3200 , Abbott, Abbott Park, Illinois, USA, www.abbott.com)

### CFU-Fs assay

Total bone marrow cells were plated into 6 wells plates, at a concentration of 5.10^5^ cells by ml of culture medium for a final volume of 3 ml in each well. The culture medium consisted of α-MEM (Cambrex, Paris, France, http://www.cambrex.com), decomplemented fetal bovine serum 10%, decomplemented horse serum 10%, l-glutamine 1%, streptomycin-penicillin 1%. The plates were maintained in an incubator at 37°C in a CO_2_ (5%) atmosphere. After 3 days, the cells were observed and the medium was changed. The colonies were stained at day 10 with May-Grunmwald Giemsa (Merck, Darmstadt, Germany, http://www.merck.be) and counted. Some cells were fixed with ethanol 70% for immunohistology and enzymatic activity studies.

### ALP activity detection

For histologic ALP activity detection, 0.5 µm JB4 embedded sections or acetone-fixed CFU-Fs colonies were incubated (60 and 15 minutes respectively at 37°C) in a reaction medium containing 1 mg/ml Fast Blue BB Salt, 0.3 mg/ml naphtol-AS-phosphate (Sigma-Aldrich, Bornem, Belgium, http://www.sigmaaldrich.com) and 0.5% NN-dimethylformamide (Sigma-Aldrich) in 0.2 M Tris Buffer (ph 9.2).

ALP activity was quantified in the cellular fraction of CFU-Fs. Cell extract (50 ml) was incubated with 100 ml of p-nitrophenylphosphate (liquid p-NPP, ready to use, KEM-EN-TEC, Kobenhavn, Denmark, http://www.kem-en-tec.com). In the presence of ALP, p-NPP is transformed to p-nitrophenol and inorganic phosphate. P-nitrophenol absorbance was measured at 405 nm after 30 min of incubation at 37°C. A standard preparation of p-nitrophenol was used for calibration. Results were expressed in nanomoles of p-nitrophenol released per min and per mg of DNA.

### Antibodies and immunohistochemistry

Mouse sdf-1, TNSALP and neuropilin-1 protein expression were assayed on CFU-Fs cultured on 20×20 mm microscopic slides and on frozen-embedded femoral bone marrow sections both fixed with cold aceton (4°).

Endogenous peroxydase activity was blocked with 3% of hydrogen peroxide in methanol at room temperature for 30 minutes. The sections were exposed for 30 min to blocking solution (Real antibody diluents, Dako cytomation, Heverlee, Belgium, http://www.dako.com) and subsequently treated with an appropriate dilution of the primary antibody.

For TNSALP, PMCA, neuropilin-1 and sdf-1 detection, cells were incubated for one hour with respectively: a polyclonal rabbit anti-TNSALP (1∶500) (A generous gift from Professor Oda, Niigata University, Niigata, Japan), a rabbit anti-PMCA antibody, (1∶200) (Santa Cruz Biotechnology Inc., Santa Cruz, Ca, http://www.scbt.com), a rabbit anti-neuropilin-1 antibody (1∶50) (BD Pharmingen, Erembodegem, Belgium, http://www.bdbiosciences.com) and a polyclonal rabbit anti-sdf-1 (1∶25) (Abcam, Cambridge, UK, http://abcam.com). This incubation was followed by an incubation of 30 minutes with a peroxydase anti-rabbit antibody (1∶200) (GE Healthcare, Diegem, Belgium, http://gehealthcare.com).

Sections were incubated with an appropriate secondary antibody (The site of immunoreaction was revealed by treating sections with a 2% DAB solution supplemented with 0.002% H_2_O_2_ (Dako Cytomation). Nuclei were counterstained with hematoxillin.

For immunofluorescence assays, sections were incubated with a monoclonal anti-rabbit Alexa 488 (Invitrogen) as a secondary antibody for 30 minutes and nuclei were counterstained with Toto 3 (1∶400) (Invitrogen).

### RNA extraction and Reverse Transcription-Polymerase Chain Reaction Analysis

Total RNA was purified from cell suspension using the High Pure RNA Isolation kit (Roche Diagnostics GMBH, Mannheim, Germany, http://www.roche-applied-sciences.com). The amount of purified RNA was quantified by measure of absorbance using the Nanodrop (ND1000, Isogen life sciences, Sint-Pieters-Leew, Belgium, http://www.isogen-lifesciences.com). The following primers were used to amplify the following endogens mRNA: endogen 28 s, 5′-GTT CAC CCA CTA ATA GGG AAC GTG A-3′ and 3′-GGA TTC TGA CTT AGA GGC GTT CAG T-5′; TNSALP, 5′-ATG GTG AGC GAC ACG GAC AAG AA -3′ and 3′-GGC ATA CGC CAT CAC ATG GGG AA -5′; osteocalcin, 5′- GAG GGC AAT AAG GTA GTG AAC A-3′and 3′- GAT GCG TTT GTA GGC GGT CTT CA -5′; runx2, 5′- ATC GCC CAC CAC CCG GCC GAA-3′ and 3′- GGC CCA CAA ATC TCA GAT CGT TGA-5′; osteopontin 5′-GAA ACT CTT CCA AGC AAT TC-3′ and 5′GGA CTA GCT TGT CCT TGT GG-3′ ; pparγ′-GGG TCA GCT CTT GTG AAT GG-3′ and 3′-CTG ATG CAC TGC CTA TGA GC-5′; neuropilin1, 5′- CAC AGT GGA ACA GGT GAT GAC TTC-3′and 3′- AAC CAT ATG TTG GAA ACT CTG ATT GT-5′; sdf-1: 5′- CT GCA TCA GTG ACG GTA AAC C-3′ and 3′- GC TTT CTC CAG GTA CTC TTG G-5′.

Reactions were performed in an automated thermal cycler (T3 thermocycler, Westburg, Leusden, Netherlands, http://www.westburg.com) using the geneAMP Thermostable rTth Reverse transcriptase RNA polymerase chain reaction (RT-PCR) kit (Roche) specific pairs of primers (Eurogentec, Liège, Belgium, http://www.eurogentec.be), and 10 ng of RNA for 25 µl of reaction mixture. Quantification analyses were made with the GelDoc EQ imaging system (Biorad, Nazareth, Belgium, http://www.bio-rad.com).

### Western immunoblotting

Cell lysates were prepared with the use of RIPA buffer (10 mM Tris [tris(hydroxymethyl)aminomethane], pH 7.4; 150 mM NaCl; 1% Triton ×−100; 0,5% deoxycholate; 0,1% sodium dodecylsulfate [SDS]; 5 mM ethylenediaminetetraacetic acid [EDTA]) containing protease inhibitors (complete tablets; Roche, Basel, Switzerland). Aliquots of protein samples (50 µg) or equivalent amount of cells (2×10^6^ cell) were mixed with the same volume of double-strenght laemmli buffer (125 mM Tris-HCl pH 6.8, 4% SDS, 20% glycerol, 10% 2-mercaptoethanol, and 0,002% bromophenol blue). The samples were boiled for 5 min and subjected to SDS-polyacrylamide gel electrophoresis (PAGE) (7-12% gradient gels). Immunoblotting was performed using monoclonal antibodies or polyclonal antisera against actin (A 2066, Sigma) Runx2 (Santa Cruz Biotechnologies) and pparγ (SantaCruz Biotechnologies). Immunodetection was performed by the using of horseradish peroxydase-conjugated secondary antibodies (rabbit IgG, horseradish peroxydase linked whole antibodies, Santacruz Biotechnologies, Santa-Cruz, CA) and an enhanced chemiluminescence method (RPN2132; Amersham, Buckinghamshire, UK) involving exposure to X-ray film (Amersham hyperfilm ECL, Buckinghamshire, UK).

### Differentiation assay

Osteoblastic differentiation was induced by culturing 10 days CFU-Fs for 5 weeks in DMEM medium (Cambrex) supplemented with fetal bovine serum (Invitrogen), l-glutamine (2.10^−3^ M), streptomycin-penicillin (1%), dexamethasone (10^−8^ M), b-glycerophosphate (10^−3^ M) and ascorbic acid (3.10^−4^ M). Cells were maintained in the inducing medium for 5 weeks with a three times a week change to overcome the instability of ascorbic acid in neutral pH. Cells were fixed for 10 minutes in 4°C ethanol (90%) and stained with an alizarin red solution (Sigma) for 5 minutes to detect hydroxyapatite deposits.

To induce adipogenic differentiation, CFU-Fs (10 days) were cultured for 3 weeks in DMEM-HG medium (Cambrex) (40%), HAM F12 medium (Cambrex) (50%) supplemented with rabbit serum (Gibco) (10%), l-glutamine (2.10^−3^ M), streptomycin-penicillin (1%), dexamethasone (10^−8^ M) and insulin (0.5 µg/ml). Cells were maintained for 3 weeks in inducing medium and then were used for lipid droplet staining using Oil-Red O (Sigma).

### Enzyme-linked immunosorbent assay

Sdf-1 protein expression was assessed on the supernatant of irradiated CFU-Fs harvested at different time points after irradiation (5 min., 10 min., 20 min., 30 min., 45 min., 1 h, 1 h30, 2 h, 3 h and 4 h). The concentration of sdf-1 was measured using an enzyme-linked immunosorbent assay (ELISA) kit (R&D Systems, Abingdon, UK, http://www.rndsystem.com) according to the manufacturer's instructions.

### Immunomagnetic separation of c-kit+Lin- cells from bone marrow

Using the lineage cell depletion kit (Miltenyi Biotec, Leiden, Netherlands, http://www.miltenyibiotec.com), negative cells were isolated from suspension of bone marrow cells by depletion of cells expressing a panel of so-called lineage antigens (CD3, CD45R/B220, CD11b, Gr-1 and Ter-119). Lineage+ cells are indirectly labeled magnetically using a cocktail of biotin-conjugated monoclonal antibodies as primary labeling reagent and anti-biotin antibodies conjugated to microbeads as secondary labeling reagent. The magnetically labeled lineage cells were removed by retaining them on their adherence to a magnetized column while the unlabeled lineage-negative cells passed through the column. The lineage-negative-cells were then incubated with a monoclonal rat anti-mouse CD117 (c-kit) antibody directly coupled with microbeads. Positive cells were enriched after separation on column from unlabelled cells that were eluted with PBS-BSA. By these two successive isolations, we obtained approximatively 10^5^ precursor cells by femur (0.51% of total hematopoietic bone marrow).

### Co-culture of CFU-Fs and C-kit+ lin- cells

Ckit+lin- cells were co-cultured on 10 days CFU-Fs from “day 3” irradiated or control mice. 2.10^5^ progenitor cells were plated in each well of a 6 wells plate in direct contact or separated from CFU-Fs by a nitrocellulose porous membrane (0.4 µm) (Nunc, Langelselbold, Germany, http://www.nuncbrand.com).

Cells were harvested at different time points of co-culture (1, 3, 5 and 7 days), counted and analyzed for proliferation, survival, colony formation and differentiation.

### Colony formation in semisolid media

A methylcellulose assay was performed to test the presence of progenitor cells. The murine ckit+lin- cells were placed in a volume of 2.5 ml of methylcellulose (Methocult GF M3434; Stem Cell technologies, Grenoble, France, http://www.stemcell.com). After 14 days of culture at 37°C and 5% CO_2_ in air, colonies consisting of more than 50 cells were counted as colony forming units (CFUs) in culture. The proportion of CFU-GM (Granulocytes-Macrophages), CFU-G (Granulocytes), CFU-M (Macrophages), BFU-E (Burst Forming Units Erythroid) and CFU-Mix (mixed granulo-erythropoïetic colony-forming units) was evaluated according to established criteria (Stem Cell technologies) using an inverted microscope.

### Flow cytometry analysis of progenitor differentiation

Hematopoietic cells harvested from co-culture were stained with biotinilated antibodies directed against lineage markers (Ter119, CD3, CD11b, Ly6-G, CD45R/B220) (Mouse lineage antibody kit, BD Pharmingen) (2 µl directly in contact with cells) for 20 min. Each aliquot was incubated with a dilution of 1/500 of steptavidin-coupled Alexa-488 (Invitrogen) for 20 min and afterward washed with PBS-BSA. Analysis was performed on a FACSCantoII (Beckton Dickinson, Immunocytometry Systems, San Jose, CA, http://www.bd.com). Specific fluorescence of Alexa488 excited at 488 nm, as well known forward and orthogonal light-scattering properties of normal murine bone marrow cells were used to establish gates. Data acquisition and analysis were performed using FACSDiva software (Beckton Dickinson).

### Flow cytometry analysis of cell survival (Annexin5 –Propidium iodide)

1.5 10^5^ haematopoietic cells harvested from co-culture were incubated for 15 minutes with 5 µl of annexin-5 and 5 µl of propidium iodide (BD Pharmingen). Analysis was performed on a FACSCantoII (Beckton Dickinson). Annexin-5 only stained apoptotic cells, whether necrotic or late apoptotic cells internalized propidium iodide. By analyzing both the fixation of annexin-5 and the internalization of PI by cells, we can distinguish living from necrotic, early apoptotic or late apoptotic cells.

### Proliferation assay (^3^H)

Exponentially growing cells were labeled with 0.5 µCi of methyl[3H]-thymidine (8 µCi/mmol, GE Healthcare, Little Chalfond, UK, http://www.ge.com) for 10 hours. Cells were lysed by a 1 M NaOH solution and 250 µl of each template was put on a 96 wells plate. Cells lysates were put on a harvester filter with a cell harvester (Unifilter 96 Harvester, Perkin Helmer, Waltham, Ma, http://www.perkinhelmer.com). Radioactivity was measured by a scintillation counter (Perkin Helmer) and expressed in count per minute (CPM).

### Statistical analysis

The results were analyzed in independent samples in two tailed and unpaired Student's t tests and are presented as mean + SE deviation (Statistica, Microsoft, http://www.microsoft.com).
